# Mobile and immobile boundaries in ferroelectric films

**DOI:** 10.1038/s41598-021-81516-w

**Published:** 2021-01-21

**Authors:** P. Yudin, K. Shapovalov, T. Sluka, J. Peräntie, H. Jantunen, A. Dejneka, M. Tyunina

**Affiliations:** 1 Institute of Physics, Academy of Sciences of the Czech Republic, Na Slovance 2, 18221 Praha 8, Czech Republic; 2 Kutateladze Institute of Thermophysics, Siberian Branch of Russian Academy of Science, Lavrent’eva av. 1, Novosibirsk, Russia; 3 Institutut de Ciència de Materials de Barcelona, ICMAB-CSIC, Campus UAB, 08193 Bellaterra, Spain; 4 CNRS, Université de Bordeaux, ICMCB, UPR, 9048, 33600 Pessac, France; 5 CREAL SA, Chemin du Paqueret 1A, CH-1025 Saint-Sulpice, Switzerland; 6 Microelectronics Research Unit, University of Oulu, P.O. Box 4500, 90014 Oulu, Finland

**Keywords:** Ferroelectrics and multiferroics, Structural properties, Actuators, Electronic devices, Information storage

## Abstract

The intrinsic mobile interfaces in ferroelectrics—the domain walls can drive and enhance diverse ferroelectric properties, essential for modern applications. Control over the motion of domain walls is of high practical importance. Here we analyse theoretically and show experimentally epitaxial ferroelectric films, where mobile domain walls coexist and interact with immobile growth-induced interfaces—columnar boundaries. Whereas these boundaries do not disturb the long-range crystal order, they affect the behaviour of domain walls in a peculiar selective manner. The columnar boundaries substantially modify the behaviour of non-ferroelastic domains walls, but have negligible impact on the ferroelastic ones. The results suggest that introduction of immobile boundaries into ferroelectric films is a viable method to modify domain structures and dynamic responses at nano-scale that may serve to functionalization of a broader range of ferroelectric films where columnar boundaries naturally appear as a result of the 3D growth.

## Introduction

Domain structures, in a large extent, determine performance of ferroelectric (FE) devices ranging from non-volatile memories to actuators and sensors^1^. With the development of nanotechnology, the demand to control individual nano-domains and domain walls (DWs) rapidly grows^2^. On the way towards reconfigurable electronics, it became possible to inject and control movement of conducting DWs in otherwise insulating materials^3^ and microscopically analyse the internal structure of individual DWs^4^. First devices based on individual DWs started to emerge^5,6^. Ambitions in the field go as far as the creation of artificial intelligence where FE tunnel junctions work as synapses^7^. A widespread implementation of FE nanoelectronics requires compatibility with existing electronic standards, giving advantage to sub-micron films grown on Si wafers^8,9^.

Fundamentals of ferroelectricity include phase transition between the higher symmetry parent phase (normally at high temperature) and the lower symmetry FE phase (at a lower temperature), which is accompanied by the appearance of spontaneous polarization. Depending on the crystal symmetry, FE polarization is bistable or multistable and can be switched by the electric field. FE domains, where the spontaneous polarization is oriented in one of the symmetry permitted directions, are separated by DWs, which are interfaces of a few unit cells in thickness. Commonly, FEs are also ferroelastics, with spontaneous strain being a secondary order parameter and depending on the polarization that is the primary order parameter. This relationship leads to a rich diversity of DW patterns^10^.

A large part of the progress in studies of FE domains was achieved with perovskite materials such as lead titanate (PTO), which are cubic in the parent phase and tetragonal in the FE phase. These materials have 6 possible polarization states and 3 states of spontaneous strain in the FE phase. Thus, two basic types of DWs are present in these materials: ferroelastic 90° DWs and non-ferroelastic 180° DWs. Behaviour of the 180° DWs are mainly governed by minimization of the electrostatic energy: their energetically preferable orientations are parallel to the polarization vector^10^. 180° DWs are typically more mobile and make an important contribution to ferroelectric switching^11^. 90° DWs are restricted by both electrostatic and mechanical compatibility conditions^10^, which essentially define a few possible energetically favorable orientations for such walls. 90° domain walls usually form the so-called c/a/c domain pattern and are less mobile, however even their small displacements contribute substantially to the electromechanical response^12^.

Nano-scale engineering of DWs allows one to achieve unique functionalities and is therefore an important research field today^13–16^. In particular, the domain configuration in epitaxial thin films depends on the orientation and symmetry of the film and the substrate, on the film-substrate mismatch between the lattice constants, and on the applied mechanical and electric stimuli^10^. A defect-free epitaxial film is a relevant idealized system, which enables the possibility to create equilibrium domain patterns using epitaxial strain^13,14,17,18^. However, perfect epitaxial growth is limited with a choice of suitable substrates and is hardly achievable for film thickness exceeding a few tens of nanometres^19^. FEs with granular microstructure are much more common, particularly in commercial use, where materials selection is restricted with cost efficiency and standardisation. The interaction of domains with and across grain boundaries is therefore of high scientific and practical interest. It was experimentally observed that the interaction of ferroelastic domains is in general stronger when crystallographic orientations in the neighbouring grains differ less from each other^20^. Specific crystallographic orientations in the neighbouring grains that allow for continuity of DW through a grain boundary were found in ceramics^21^. Interaction of an individual DW with various grain boundaries in thin films was experimentally explored in the work^22^, where an intriguing result was obtained: grain boundaries characterized by large tilt angles hampered motion of DWs, whereas low-angle grain boundaries unexpectedly enhanced DW motion in the vicinity of the boundary. The experimental findings suggest a crucial role of low-angle boundaries for DW behaviour, which requires a comprehensive analysis.

Here we present the results of phase-field modelling of domain patterns and DW dynamics in sub-micron thick cube-on-cube-type (001) epitaxial FE films containing low-angle columnar boundaries (CBs). The columnar structure, where columnar grains are all of nearly the same crystallographic orientation, was evidenced experimentally and considered in the simulations. The Landau-Ginsburg-Devonshire parameters and elastic properties for the model are set to represent properties of cubic-to-tetragonal ferroelectric—ferroelastic Pb(Zr_0.1_Ti_0.9_)O_3_ (PZT). The study focuses on the interaction of ferroelastic and non-ferroelastic domain walls with CBs. Particularly, we show why ferroelastic domains can propagate beyond CBs, and investigate the impact of CBs on ferroelectric switching. The numerical results on the domain structure and switching are compared with multiple available experimental observations.

## Results and discussion

The simulations that we perform aim at describing ferroelectric behaviour in cube-on-cube-type epitaxial (001) oriented thin films grown in a Stranski–Krastanov (SK) mode, where CBs result from a transition from a layer-by-layer growth to an island-type growth with increasing film thickness. Since the same epitaxial relationship holds in all columnar grains, CBs represent low-angle twin boundaries with possible stacking faults (supplementary materials C). To study these objects, we implemented a 2D phase field model of PZT 10/90, solving equations of elasticity, Gauss law, and Landau-Ginsburg-Devonsire (LGD) equations for the ferroelectric polarization self-consistently (supplementary materials A). Previously, versions of the model passed multiple tests by comparing experimental data for barium titanate (see e.g.^23^), and in particular PZT 10/90 (see e.g.^13^) epitaxial thin films. Here we introduced CBs in the model as fixed lines, whose distributions and shapes come from SK-growth. Zero polarization was set at CBs in accordance with their expected non-FE nature, which is in line with observations of suppressed piezoresponses at CBs^24–26^. This condition is equivalent to the introduction of a dead layer of nominally zero thickness, but, due to the correlation interaction, CBs have an effective thickness of the order of the correlation length (the same as that of the 180-degree DWs).

**Figure 1:**
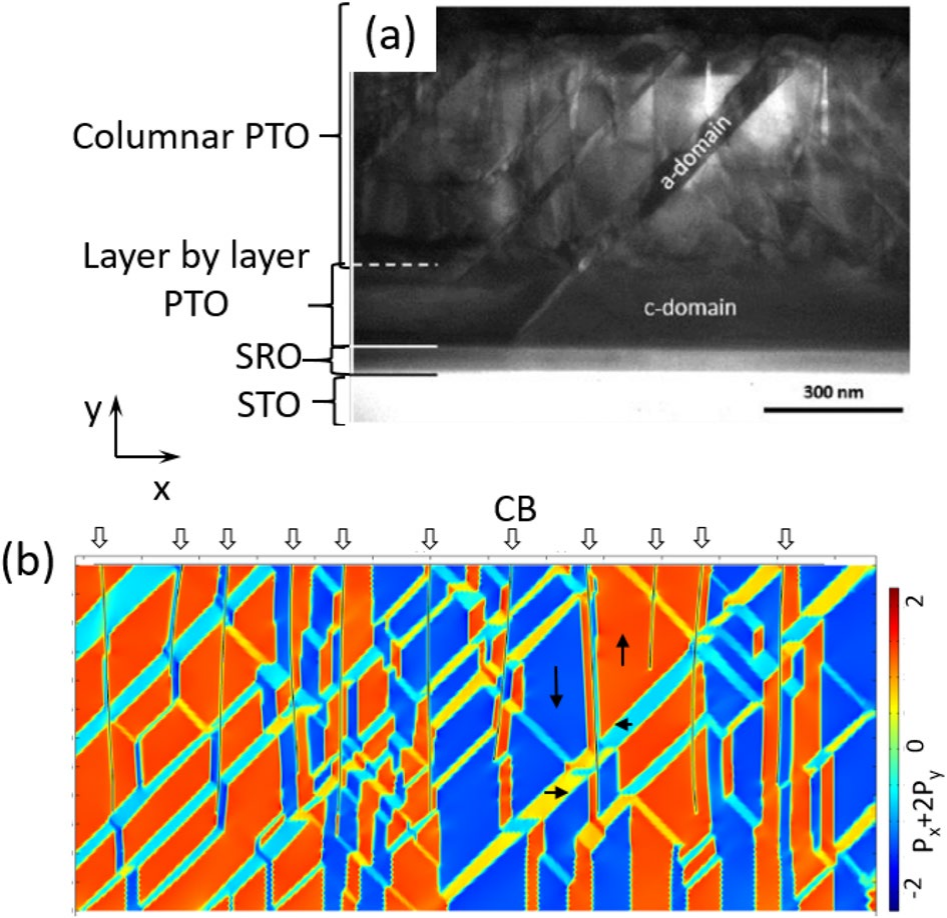
Domains in the columnar thin film. (**a**) Low-magnification cross-sectional transmission electron microscopy image in [110] direction. (**b**) Phase field simulation (c-domains are blue and red, a-domains are yellow and turquoise).

The phase field model was tested by comparison with the detected columnar and domain structures in epitaxial 600-nm-thick PbTiO_3_ (PTO) films deposited on (001) SrTiO_3_ (STO) substrate using bottom SrRuO_3_ (SRO) layer (Fig. 1, supplementary materials C, and “Methods”). The comparison between PZT 10/90 model and real PTO films is justified by the closeness of their compositions and therefore of their properties, such as polarization, correlation length and dynamic behaviour. In Fig. 1a, the a-domains are seen as stripes inclined at approximately 45° to the substrate surface, that corresponds to the mechanically compatible orientations of these domains^10^. The volume fraction of the a-domains is ~ 15%.

The widths of the experimentally observed a-stripes are smaller than or equal to the widths of columns, but the lengths extend over several columns. The a-domains are arranged in a way typical for column-free a/c polydomain epitaxial FE films of similar thickness^27^. The ferroelastic domain formation is readily interpreted in terms of stress release. The fraction of a-domains $\alpha$ may be estimated using the mean strain approach^28^

1 $$\alpha = \left( {1 + \nu } \right)\frac{{a_{s} - a}}{c - a},$$

where $\nu \approx$ 0.3 is the Poisson ratio, $a$ and $c$ are short and long dimensions of the tetragonal unit cell of the film, and ${a}_{s}$ is the lattice constant of the cubic substrate. For the parameters of epitaxially grown PTO on STO (${a}_{s}=0.3905$ nm^29^, $a=0.39045$, $c=0.41524$^30^), Eq. (1) gives a fraction of the a-domains 0.2%. However, because of the misfit release due to CB formation at high temperatures, the film should behave as if it were grown on a substrate with larger lattice constant (see supplementary materials C). The experimentally observed domain fraction $\alpha$~ 15% may be readily interpreted using a larger effective substrate lattice constant ${a}_{s}=0.3933$ nm. We made the corresponding correction of the lattice constant in our phase field model, taking also into account the slight difference of parameters between PZT and PTO. Figure 1b illustrates the cross-sectional view of a typical simulated polarization vector distribution, which reproduces the main experimental observations. Remarkably, both the experimentally detected and the simulated a-domains extend coherently through multiple columns, and the CBs do not essentially disturb a-domains.

Additionally, Fig. 1b illustrates the spatial arrangement of non-ferroelastic 180° DWs, that we observe to be strongly interacting with the CBs in our simulations. This behaviour is in agreement with piezo-force microscopy (PFM) studies, which indicate that 180° DWs almost always coincide with CBs in the SK-type columnar ferroelectric films^24,25,31^. In our phase field simulations, these DWs do prefer to coincide with CBs, although not always stick to them (Fig. 1b). This minor disparity likely originates from a non-negligible impact of the PFM tip causing some DW motion towards the CB during the measurements. However, both the theoretical and experimental results clearly evidence the attraction of 180° DWs to CBs. Below, we further rationalize the observed behaviours of both 90° and 180° DWs, addressing their interaction with CBs numerically by phase field modelling and also analytically.

**Figure 2:**
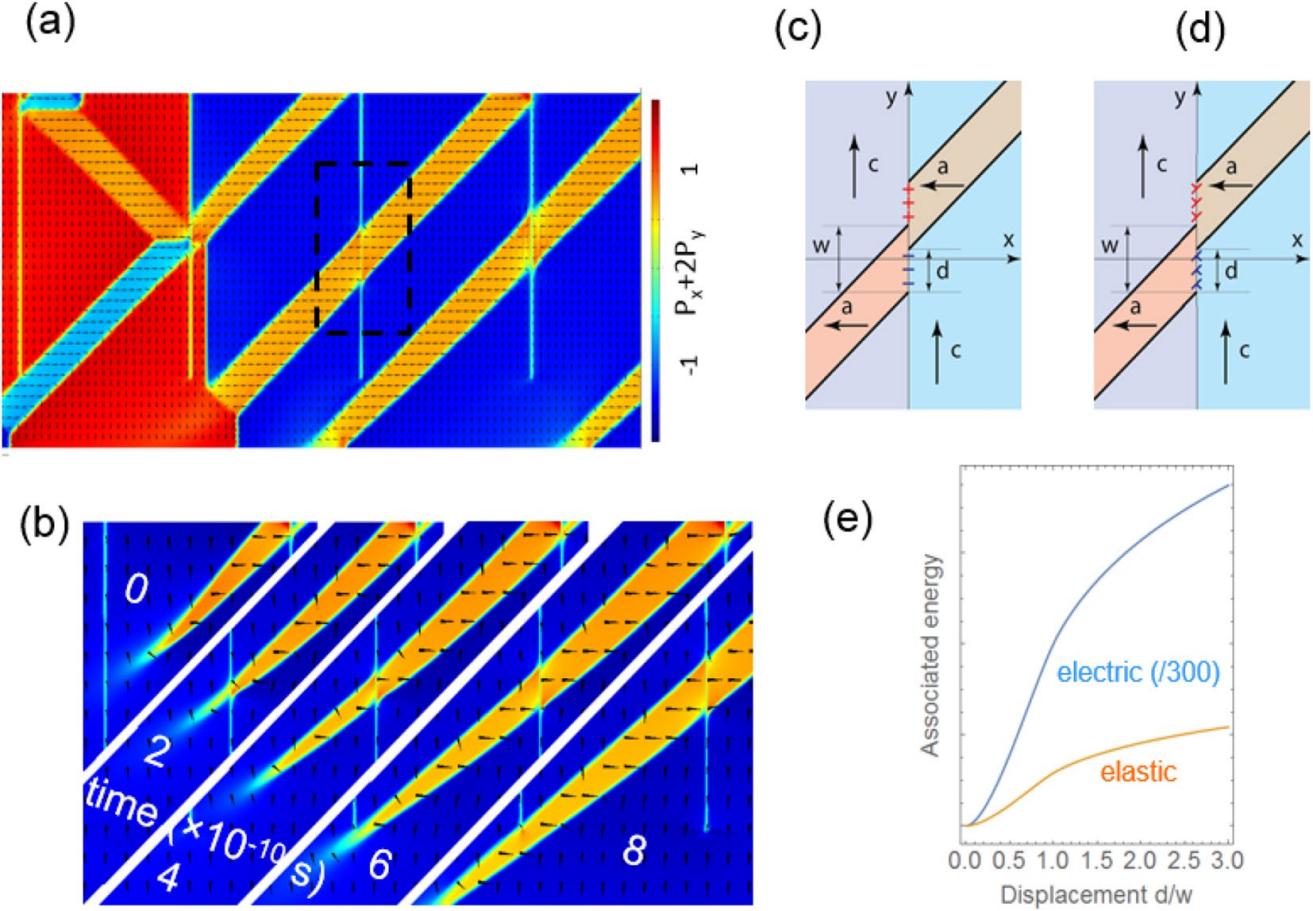
Ferroelastic domains and their interaction with grain boundaries. (**a**) Domain structure calculated using phase field model. (**b**) Five consequent frames (time shown with white font) for the time-dependent simulation showing a-domain growth through a CB, (**c**), (**d**) electrostatic and elastic representation for the domain shift at a CB, showing equivalent charge and fictitious dislocations respectively. (**e**) Electrostatic and elastic energy penalties associated with the a-domain shift d (electric energy reduced by factor 300 to imitate screening).

To explore the interaction between ferroelastic DWs and CBs, we performed a focused theoretical study using phase field and fictitious dislocation models. Phase field modelling was run for 200-nm-thick films with straight CBs. The enlarged picture of the domain structure is shown in Fig. 2a, where one can see that domains propagate continuously through CBs (right-hand side of Fig. 2a), whereas more complex crossings involving 180° DWs may also appear (left-hand side of Fig. 2a).

Next, we focussed on the simple crossing of an a-domain with CB and inspected dynamics of domain formation. We introduced a single a-domain nucleus near the surface, and observed its growth driven by minimisation of tensile strain in the thin film during a time-dependent simulation. The results are presented in Fig. 2b, where the five consequent images (from top left to right bottom) show the growth dynamics for the knife-shaped a-domain. Remarkably, CBs are almost "transparent" for the growing a-domain as they do not exert any visible resistance.

To scrutinize the effect of CBs on the a-domain, we derive estimates for the energy penalty in case where two parts of the a-domain in the neighbouring columns are shifted along the CB. The shift leads to energy penalties. Electrostatic energy penalties originate from the appearance of non-compensated bound charges. Screening will result in a reduction of electrostatic energy, which will strongly depend on type of carriers and their concentrations in the regions where $\mathrm{div}\mathbf{P}\ne 0$ (Fig. 2c). Here we assume the electrostatic energy may be reduced with some factor with respect to the case where no screening takes place. Careful analysis of the energetics in the presence of mobile charges is beyond this work. Elastic energy penalties arise because the boundary between a- and c-domains becomes mechanically incompatible. Electrostatic energy is calculated from Coulomb interaction between the bound charges. The fictitious dislocation approach is employed for the elastic energy^32,33^. In this approach, the elastic strains and stresses that arise in c/a/c domain patterns, can be derived as originating from fictitious edge dislocations, which are located at the boundaries between regions with different spontaneous strains^32–35^. The dislocation representation for the problem addressed here is shown in Fig. 2d. We obtain the following expression for the excess energy (see Supplementary Materials B):

2 $$W={W}_{elast}+{W}_{elec}=\left(\frac{E{\epsilon }_{T}^{2}}{4\pi \left(1-{\nu }^{2}\right)}+\frac{\gamma {P}_{S}^{2}}{2{\varepsilon }_{b}}\right)\left({w}^{2}\mathrm{ln}\frac{{w}^{2}-{d}^{2}}{{w}^{2}}+2wd\mathrm{ln}\frac{w+d}{w-d}+{d}^{2}\mathrm{ln}\frac{{w}^{2}-{d}^{2}}{{d}^{2}}\right),$$

where ${\varepsilon }_{b}$ is the background dielectric permittivity, $\gamma < 1$ is a factor accounting for screening of the bound charge, ${\epsilon }_{T}=(c-a)/a$ is the tetragonality factor, *E* is the Young’s modulus, ${P}_{S}$ is the spontaneous polarization, *w* is the domain width, *d* is the vertical shift (see Fig. 2c,d). The solution, Eq. (2) is illustrated in Fig. 2e. For small vertical shift *d,* the energy scales as ${d}^{2}$. This dependence explains why the a-domain prefers to propagate through CBs continuously, without shifts. In summary, this analytical treatment highlights that ferroelastic domains strongly interact through elastic and electric fields even when these domains belong to different columns. As a consequence, a-domains prefer to align and form one “super-domain” spanning several columns.

**Figure 3:**
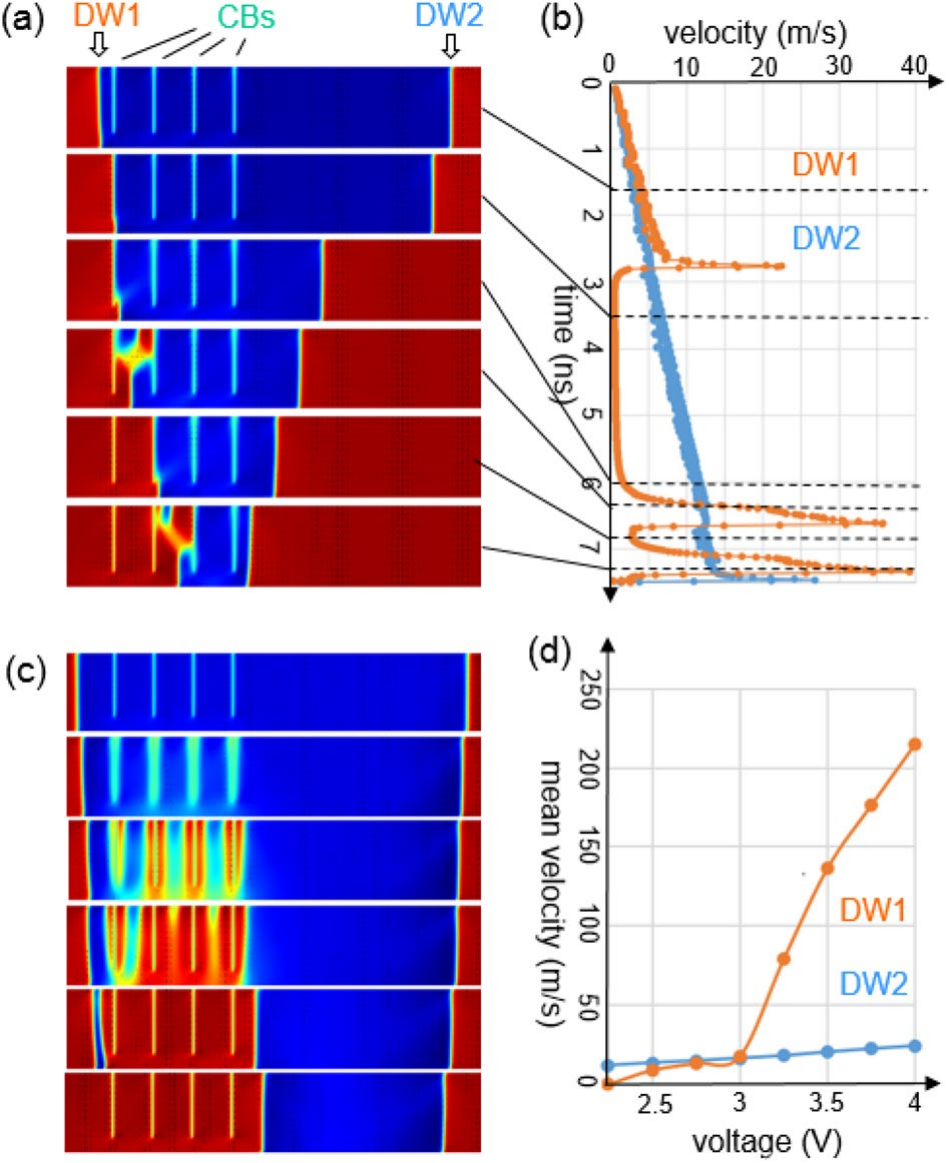
180-degree domain walls and their interaction with columnar boundaries. (**a**), (**b**) Ferroelectric switching with voltage linearly with time from 0 to 3 V. (**a**), (**d**) Switching at constant voltage, (**c**) 4 V, (**d**) DW mean speed on voltage dependence. Film thickness 20 nm.

Next, to explore the interaction of non-ferroelastic 180° DWs with CBs, we ran a series of phase field simulations for films with a thickness of 20 nm, which provided a high enough resolution of DW regions. Figure 3 studies the motion of 180° DWs under a continuous top electrode during FE switching. For the case of Fig. 3a,b, voltage linearly increases with time. The motions of two DWs are compared: DW1 passing through a set of CBs (left-hand side) and DW2 moving without obstacles (right-hand side). Velocities of the two DWs versus time are evaluated from the mean polarization in the corresponding part of the film, and plotted in Fig. 3b. The speed of the "freely moving" DW2 is proportional to the applied voltage as expected for a low-field wall mobility in a non-activated regime^10^. The motion of DW1 is much more complicated. DW1 starts with the same speed as that of DW2. When DW1 approaches the first CB, it first accelerates and then sticks to CB1. Such behaviour confirms that there is an attraction between the CBs and 180° DWs. Further, DW1 stays immobile at the CB for a wide range of applied voltages, while DW2 constantly advances. When a critical voltage of 2.25 V is reached, DW1 detaches from CB1. It moves in a hopping manner, jumping from one CB to another CB. Its speed during a jump is much higher than that of the free-moving DW2, partially due to the contribution of the additional DW nucleating at the top of the CB in front (4th and last images of Fig. 3a). However, between the jumps, the effect of pinning still holds, slowing the DW1 down at each CB. The resulting average speeds of DW1 and DW2 are therefore comparable in the voltage range 2.5–3 V. We note that in practice, there often exists a threshold electric field for DW motion^36^, which is zero in our case. DW speeds are determined by the balance of the driving force associated with the electric field on one hand and dissipative forces, which can be generally decomposed to “dry” and “viscous” friction mechanisms on the other hand^37^. Here the threshold electric field is null, as we neglect the “dry friction” mechanisms and take into account only “viscous” term. In our model the speeds of all processes scale linearly with the kinetic coefficient Γ, which we choose two orders of magnitude smaller than the one associated with the soft-mode frequency^10^, see Supplementary Materials A.

Further we run a series of numerical experiments with the same initial and boundary conditions, but with different applied voltages, which are kept constant during each individual simulation. Average speeds of propagation of DW1 and DW2 are compared as functions of the voltage in Fig. 3d. Overall, we observe two effects of CBs on DW1: pinning and nucleation, depending on the applied voltage. The pinning effect completely prevents movement of DW1 through CBs for voltages below 2.25 V. With further increase of voltage, the pinning effect diminishes and vanishes at around 3 V, with the speed of propagation of DW1 comparable to that of DW2. Further increase of voltage leads to the appearance of nucleation effect. In the voltage range 3–4 V, we observe intensive nucleation of DWs at each CB. The speed of nucleation increases with the increase of voltage, resulting in a much faster switching of the region containing CBs in comparison to the region without CBs (Fig. 3c). In the voltage range below 3 V, DW nucleates at CB only in front of another DW, approaching the CB (4th and last images in Fig. 3a). At voltages exceeding 4 V, the film even without CBs contains large areas where polarization switches almost instantaneously. However, this fast switching regime is beyond the scope of the present work.

A conclusion can be drawn that CBs are apt to suppress switching at low voltages and enhance switching at high voltages. A hopping regime of switching, where a DW “jumps” from one CB to another, is predicted for intermediate voltages. Our theoretical results also suggest that CBs are important centres for the nucleation of new 180° DWs. The presented 2D model is valid for FE switching under continuous top electrodes. However, one can expect that our main conclusions also hold for the case of PFM switching. Our numerical simulations are consistent with the PFM observation of the 180° DWs in columnar films: the DWs almost always coincide with CBs^24–26,31^, a single domain typically embraces a few columns, and a DW can propagate several columns beyond the area where switching voltage was applied to^25^.

The above results may be elucidated in terms of the energy of interaction between CBs and 180° DWs. The 180° DWs that we study in this work are Ising-type, i.e., the polarization in their centre is zero. Because the polarization is also absent in CBs, a 180° DW is largely energetically subsidized when passing through a CB. Namely, for the conditions of Fig. 3, we measured the energy density of a single DW to be 0.2 ± 0.02 J/m^2^, the energy of a CB—0.19 ± 0.01 J/m^2^, and the energy of the pair (DW attached to a CB)—0.19 ± 0.02 J/m^2^. This means that the DW has nearly zero energy when passing through a CB whose orientation coincides with that of an electrically compatible DW (vertical for domain polarisations up and down). For inclined CBs, the energy of a DW at the CB is expected to be similar to the energy of electrostatic incompatibility associated with the inclination. The subsidized DW energy on a CB explains both the halt of a moving DW at a CB and nucleation of new DWs at CBs. Our hypothesis of zero polarization at CB is in accordance with a suppression of the piezoresponse around CBs in SK-columnar films^24–26^. Unfortunately, quantitative verification is inaccessible by PFM because of effects of surface morphology and lateral resolution. The complete suppression of the polarization at CBs was chosen in our model in accordance with the PFM observations of 180° DWs in the columnar films, where DWs strongly apt to attach to CBs^24–26,31^. We expect that our results will hold qualitatively for the case of a “partial polarization suppression” if they also take place in real systems. A further refinement to the theory may be envisaged from the results of Ab Initio simulations^38^, where PTO DW energy is predicted to be 0.13 or 0.17 J/m^2^, depending whether the wall is centred on Ti or Pb atoms. Because CBs are not likely to be centred on Ti or Pb atoms, a relatively higher energy is expected for DW attached to a CB. In the case of zero-polarization condition on a CB, we expect the energy subsidy for a DW on a CB to change from 100 to 80–90% of the nominal DW energy taking into account the results of Ref.^38^.

In summary, ferroelastic domains are shown to only weakly interact with CBs, which explains their continuity through CBs. A discontinuity would lead to an energy penalty, for which we obtain approximate analytical expression. Because the same epitaxial relationship holds in all columns, the electric and elastic couplings between ferroelastic domains explain their alignment between columns. Continuous ferroelastic “super-domains” are formed: they span several columns and behave as in a defect-free epitaxial film. In contrast, a strong interaction of nonferroelastic 180° DWs with CBs is demonstrated. This is rationalized by the fact that polarization passes through zero in the middle of both CBs and DWs, so that the DW energy is almost zero when it attaches to a CB. The modelling results have important implications for FE switching. 180° DWs halt at CBs, which impedes switching for low applied voltages. On balance, when large enough bias is applied, CBs work as nucleation centres, leading to enhanced switching. Having these unique properties, ferroelectric systems with as-grown CBs demonstrate potential for both nanoelectronic applications, and for achievement of superior macroscopic properties. Taking into account that columnar films can be grown on a much broader range of substrates and to larger thicknesses than pure epitaxial films, the results have far-reaching applied value.

## Methods

The results of the phase-field model were obtained by the numerical solution of two-dimensional version of the time-dependent Landau–Ginzburg–Devonshire equation^39,40^, Poisson’s equation for dielectrics and equation of motion in elastic material as described in Supplementary Materials A, with material parameters corresponding to PZT (Zr:Ti = 10:90) from Refs^29,30,41–46^. The model is numerically solved by the finite element method with a time-dependent solver in COMSOL 5.3. The fictitious dislocation model used to rationalize the results is described in Supplementary Materials B.

The PbTiO_3_ films were prepared by pulsed laser deposition on (001) SrTiO_3_ substrates. The growth details can be found in Ref^47^. XRD measurements were performed on a Bruker 8 diffractometer using Cu Kα radiation. Cross-sectional samples were prepared by focused ion beam (FIB) milling using a Dual-Beam scanning electron microscope FEI Helios Nanolab 600 equipped with a Ga + FIB. The cross-sections were inspected by a transmission electron microscopy using a JEOL JEM-2200FS and FEI Helios Nanolab Dualbeam microscopes. More details in Supplementary materials C.

## Supplementary information

Supplementary Information.

## Data Availability

The authors indicate that the generated and analyzed data that support the findings of this study are available within the paper and Supplementary Information.

## References

[1] Dawber M, Rabe K, Scott J (2005). Physics of thin-film ferroelectric oxides. Rev. Mod. Phys..

[2] Catalan G, Seidel J, Ramesh R, Scott JF (2012). Domain wall nanoelectronics. Rev. Mod. Phys..

[3] McQuaid RG, Campbell MP, Whatmore RW, Kumar A, Gregg JM (2017). Injection and controlled motion of conducting domain walls in improper ferroelectric Cu-Cl boracite. Nat. Commun..

[4] Cherifi-Hertel S (2017). Non-Ising and chiral ferroelectric domain walls revealed by nonlinear optical microscopy. Nat. Commun..

[5] Sharma P (2019). Conformational domain wall switch. Adv. Func. Mater..

[6] Sharma P (2017). Nonvolatile ferroelectric domain wall memory. Sci. Adv..

[7] Boyn S (2017). Learning through ferroelectric domain dynamics in solid-state synapses. Nat. Commun..

[8] Muralt P (2000). Ferroelectric thin films for micro-sensors and actuators: a review. J. Micromech. Microeng..

[9] Setter N (2006). Ferroelectric thin films: review of materials, properties, and applications. J. Appl. Phys..

[10] Tagantsev A, Cross L, Fousek J (2010). Domains in ferroic crystals and thin films.

[11] Taylor D, Damjanovic D (1997). Evidence of domain wall contribution to the dielectric permittivity in PZT thin films at subswitching fields. J. Appl. Phys..

[12] Damjanovic D (1997). Logarithmic frequency dependence of the piezoelectric effect due to pinning of ferroelectric-ferroelastic domain walls. Phys. Rev. B.

[13] Feigl L (2014). Controlled stripes of ultrafine ferroelectric domains. Nat. Commun..

[14] Nesterov O (2013). Thickness scaling of ferroelastic domains in PbTiO3 films on DyScO3. Appl. Phys. Lett..

[15] Sung JH (2013). Single ferroelectric-domain photovoltaic switch based on lateral BiFeO_3_ cells. NPG Asia Mater..

[16] Karpov D (2017). Three-dimensional imaging of vortex structure in a ferroelectric nanoparticle driven by an electric field. Nat. Commun..

[17] Catalan G (2011). Flexoelectric rotation of polarization in ferroelectric thin films. Nat. Mater..

[18] Khan AI, Marti X, Serrao C, Ramesh R, Salahuddin S (2015). Voltage-controlled ferroelastic switching in Pb (Zr0.2Ti08) O_3_ thin films. NanoLett..

[19] Ohring M (2002). Materials science of thin films, 2nd edn.

[20] Ivry Y, Chu D, Scott JF, Durkan C (2011). Domains beyond the grain boundary. Adv. Funct. Mater..

[21] Mantri S, Oddershede J, Damjanovic D, Daniels JE (2017). Ferroelectric domain continuity over grain boundaries. Acta Mater..

[22] Marincel DM (2015). Domain wall motion across various grain boundaries in ferroelectric thin films. J. Am. Ceram. Soc..

[23] Sluka T, Tagantsev AK, Bednyakov P, Setter N (2013). Freeelectron gas at charged domain walls in insulating BaTiO 3. Nat. Commun..

[24] Plekh M, Narkilahti J, Levoska J, Tyunina M (2010). Polydomain configuration in epitaxial Pb0.5Sr0.5TiO3/La0.5Sr0.5CoO3 heterostructures. Appl. Phys. Lett..

[25] Plekh M, Tyunina M (2010). Ferroelectric domains in epitaxial PbZr 065 Ti 0.35 O 3/La 05 Sr 0.5 CoO 3 heterostructures. Appl. Phys. Lett..

[26] Tyunina M, Yao L, Plekh M, Levoska J, van Dijken S (2013). Epitaxial ferroelectric heterostructures with nanocolumnenhanced dynamic properties. Adv. Funct. Mater..

[27] Ganpule C (2000). Role of 90 domains in lead zirconatetitanate thin films. Appl. Phys. Lett..

[28] Roytburd, A. L. Elastic domains in ferroelectric epitaxial films. In *Thin film ferroelectric materials and devices*, 71–90 (Springer, Berlin, 1997).

[29] Okazaki A, Kawaminami M (1973). Lattice constant of strontium titanate at low temperatures. Mater. Res. Bull..

[30] Landolt, H. & Bornstein, R. *Numerical data and functional relationships in science and technology: Astronomy, astrophysics and space research. New series. Group 6*, vol. 1 (Springer, 1965).

[31] Andreeva N, Emelyanov AY (1997). Low-temperature evolution of local polarization properties of PbZr0. 65Ti035O3 thin films probed by piezoresponse force microscopy. Appl. Phys. Lett..

[32] Pertsev N, Emelyanov AY (1997). Stability diagram for elastic domains in epitaxial ferroelectric thin films. Phys. Solid State.

[33] Romanov A (1998). Domain pattern formation in epitaxial rhombohedral ferroelectric films. II Interfacial defects and energetics. J. Appl. Phys..

[34] Pompe W, Gong X, Suo Z, Speck J (1993). Elastic energy release due to domain formation in the strained epitaxy of ferroelectric and ferroelastic films. J. Appl. Phys..

[35] Pertsev N, Zembilgotov A (1995). Energetics and geometry of 90 domain structures in epitaxial ferroelectric and ferroelastic films. J. Appl. Phys..

[36] Shur VY, Esin A, Alam M, Akhmatkhanov A (2017). Superfast domain walls in KTP single crystals. Appl. Phys. Lett..

[37] Yudin P, Hrebtov MY, Dejneka A, McGilly L (2020). Modeling the motion of ferroelectric domain walls with the classical Stefan problem. Phys. Rev. Appl..

[38] Meyer B, Vanderbilt D (2002). Ab initio study of ferroelectric domain walls in PbTiO 3. Phys. Rev. B.

[39] Li Y, Chen L (2006). Temperature-strain phase diagram for BaTiO3 thin films. Appl. Phys. Lett..

[40] Semenovskaya S, Khachaturyan A (1998). Development of ferroelectric mixed states in a random field of static defects. J. Appl. Phys..

[41] Haun M, Zhuang Z, Furman E, Jang S, Cross LE (1989). Thermodynamic theory of the lead zirconate-titanate solid solution system, part III: Curie constant and sixth-order polarization interaction dielectric stiffness coefficients. Ferroelectrics.

[42] Pertsev N, Kukhar V, Kohlstedt H, Waser R (2003). Phase diagrams and physical properties of single-domain epitaxial Pb (Zr1-xTix) O 3 thin films. Phys. Rev. B.

[43] Li Y, Hu S, Liu Z, Chen L (2002). Effect of substrate constraint on the stability and evolution of ferroelectric domain structures in thin films. Acta Mater..

[44] Hlinka J, Ondrejkovic P, Marton P (2009). The piezoelectric response of nanotwinned BaTiO3. Nanotechnology.

[45] Tagantsev AK (2008). Landau expansion for ferroelectrics: which variable to use?. Ferroelectrics.

[46] Shirane G, Suzuki K, Takeda A (1952). Phase transitions in solid solutions of PbZrO3 and PbTiO3 (II) X-ray study. J. Phys. Soc. Jpn..

[47] Perantie J, Stratulat M, Hannu J, Jantunen H, Tyunina M (2016). Enhancing polarization by electrode-controlled strain relaxation in PbTiO3 heterostructures. APL Mater..

