# Supplementary material for: Mobile and immobile boundaries in ferroelectric films
Source: Sci Rep. 2021 Jan 21;11:1899. doi: 10.1038/s41598-021-81516-w (PMC7820330; doi:10.1038/s41598-021-81516-w)
Supplement: Supplementary file 1 — Supplementary Information. [file 41598_2021_81516_MOESM1_ESM.docx]

supplementary materials

to

**Mobile and immobile boundaries in ferroelectric films**

**P. Yudin, K. Shapovalov, T. Sluka, J. Peräntie, H. Jantunen, A. Dejneka, and M. Tyunina.**

**A: The phase-field model**

| Parameter | Value | Unit | Ref. |
| --- | --- | --- | --- |
| *α*_1_ | (*T* – 741.15)3*.*6504 × 10^5^ | *Jm/C*^2^ |  |
| *α*_11_ | -5.845× 10^7^ | *Jm*^5^*/C*^4^ |  |
| *α*_12_ | 7.063 × 10^7^ | *Jm*^5^*/C*^4^ | [41] |
| *α*_111_ | 2.518 × 10^8^ | *Jm*^9^*/C*^6^ |  |
| *α*_112_ | 8.099 × 10^8^ | *Jm*^9^*/C*^6^ |  |
| *α*_123_ | -4*.*359 × 10^9^ | *Jm*^9^*/C*^6^ |  |
| *c*_11_  *c*_12_  *c*_44_ | 1.7042 × 10^11^  7*.*60803× 10^10^  8*.*333 ×10^10^ | *J/m*^3^  *J/m*^3^  *J/m*^3^ | [41,42]* |
| *q*_11_  *q*_12_  *q*_44_ | 1*.*06817 × 10^10^ 3.04×10^8^ 5*.*475×10^9^ | *Jm/C*^2^ *Jm/C*^2^ *Jm/C*^2^ | [41,42]* |
| *G*_11_  *G*_12_  *G*_44_ | 4.14048×10^-10^ 0 2*.*07×10^-10^ | *Jm*^3^*/C*^2^ *Jm*^3^*/C*^2^ *Jm*^3^*/C*^2^ | [43]* |
| Γ | 4 × 10^2^ | *C*^2^*/*(*Jms*) | [44]** |
| *ε_B_* | 10 | 1 | [45]*** |
| $a^{PZT}\vert_{T=300K}$  $r^{PZT}$ | 0,3961  8.73×10^-6^ | *nm*  *K^-1^* | [30]  [46] |
| $a^{STO}\vert_{T=300K}$  $r^{STO}$ | 0,3905  9.4×10^-6^ | *nm*  *K^-1^* | [29] |

**Supplementary Table S1**: Values of material coefficients for Pb(Zr_0,1_Ti_0,9_)O_3_ and STO used in the simulations.

*****-calculated from…;**-selected two orders of magnitude lower than … ;

***-upper estimate from…

The phase-field simulation incorporates coupling between ferroelectric and elastic properties. Model equations are obtained by the Lagrange principle from Helmholtz free energy density

[39]:

$f\left[ \left\{ P_{i},P_{i,j},e_{i,j}, D_{i} \right\} \right]=f_{bulk}^{\left( e \right)}+f_{ela}+f_{es}+f_{grad}+f_{ele}$, (S1)

where *P_i_* is the ferroelectric part of polarization, *P_i,j_* its derivatives (the subscript ’ *_,i_*’ represents the operator of spatial derivatives *∂/∂x_i_*), *D_i_* the electric displacement and *e_ij_* = 1*/*2(*u_i,j_* + *u_j,i_*) is the elastic strain where *u_i_* is a displacement vector.

The bulk free energy density

${f_{bulk}^{\left( e \right)} \left[ \left\{ \boldsymbol{P} \right\} \right]=\alpha}_{1} \sum_{i} P_{i}^{2} + \alpha_{11}^{\left( e \right)}\sum_{i} P_{i}^{4} +\alpha_{12}^{\left( e \right)}\sum_{i>j} P_{i}^{2}P_{j}^{2}+$

$\alpha_{111}\sum_{i} P_{i}^{6} +\alpha_{112}\sum_{i>j} (P_{i}^{4}P_{j}^{2}+P_{j}^{4}P_{i}^{2})+\alpha_{123}\prod_{i} P_{i}^{2}$ (S2)

is expressed for a zero strain as a six-order polynomial expansion [39] where $\alpha_{i},\alpha_{ij}^{\left( e \right)},\alpha_{ijk}$ are parameters fitted to the crystal properties (see Supplementary Table **S1** and expressions for $\alpha_{ij}^{\left( e \right)}$ below). The remaining contributions represent densities of elastic energy $f_{ela}[\{e_{ij}\}] = 1/2 c_{ijkl}e_{ij}e_{kl}$, where $c_{ijkl}$ is the elastic stiffness; electrostriction energy $f_{es}[\{P_{i},e_{ij}\}] = -q_{ijkl}e_{ij}P_{k}P_{l}$, where $q_{ijkl}$ are the electrostriction coefficients; gradient energy $f_{wall}[\{P_{i,j}\}] = 1/2G_{ijkl}P_{i,j}P_{k,l}$, where $G_{ijkl}$ are the gradient energy coefficients; and electrostatic energy *f*_ele_[{*P_i_,D_i_*}] = 1*/*(2*ε*_0_*ε_B_*)(*D_i_* −*P_i_*)^2^, where *ε*_0_ and *ε_B_* are permittivity of vacuum and relative background permittivity, respectively. The zero-strain coefficients $\alpha_{ij}^{\left( e \right)}$ can be expressed in terms of the usually introduced stress-free coefficients *α_ij_* as follows:

$$\alpha_{11}^{(e)} = \alpha_{11} +\frac{1}{6}\left( \frac{2\left( q_{11} - q_{12} \right)^{2}}{c_{11} - c_{12}}+\frac{{{(q}_{11} + 2q_{12})}^{2}}{c_{11} + 2c_{12}} \right),$$

$$\alpha_{12}^{(e)} = \alpha_{12} +\frac{1}{6}\left( \frac{2\left( q_{11}+2 q_{12} \right)^{2}}{c_{11}+ {2c}_{12}}-\frac{2{{(q}_{11}- q_{12})}^{2}}{c_{11}- c_{12}} \right)+\frac{3q_{44}^{2}}{4c_{44}}).$$

By using the Legendre transformation to electric enthalpy

*h*[{*P_i_,P_i,j_,u_i,j_,φ_,i_*}] = *f*[{*P_i_,P_i,j_,e_ij_,D_i_*}] − *D_i_E_i_,*

where *E_i_* = −*φ_,i_* is the electric field and *φ* the electric potential. By using the Lagrange principle, we can uniformly express the set of field equations which govern the kinetics of ferroelectrics:

$\left( \frac{\partial h}{\partial e_{ij}} \right)_{,j}=0,$ (S3)

$\left( \frac{\partial h}{\partial E_{i}} \right)_{,i}=0,$ (S4)

$\frac{1}{\Gamma}\frac{\partial P_{i}}{\partial t}- \left( \frac{\partial h}{\partial P_{i,j}} \right)_{,j}=\frac{\partial h}{\partial P_{i}}$. (S5)

Equation (S3) defines the mechanical equilibrium while inertia is neglected. Equation (S4) represents Gauss’s law of dielectrics. Equation (S5) is the time dependent Landau-Ginzburg-Devonshire equation [40], which governs the spatiotemporal evolution of spontaneous polarization with kinetics given by coefficient Γ. The values of the simulation parameters are introduced in Tab. **S1**.

The model geometry consists of a 20-600 nm thick block of (001) oriented PZT (Zr:Ti=10:90) that is mechanically coupled on the bottom side with at least a 5 times thicker block of STO substrate. The STO is governed only by the elastic equilibrium Eq. (S3), where for simplicity the elastic properties are set the same as in PZT. The lattice mismatch between the film and substrate is introduced by setting an artificial in-plane spontaneous strain e_s_ to STO according to the lattice parameters difference:

$e_{s} =\frac{a^{STO}(1 + r^{STO}\left( T-300) \right)}{a^{PZT}(1 + r^{PZT}\left( T-300 \right))}$ , (S6)

where $a^{STO}$ and $a^{PZT}$ are the room temperature lattice constants and $r^{STO}$and $r^{PZT}$the thermal expansion coefficients of STO and PZT, respectively.

The film and substrate have defined periodic boundary conditions with a period in the horizontal direction at least 5 times greater than the film thickness. The block of PZT is mechanically free (has zero out-of-plane stress) on the upper surface and the substrate bottom surface is mechanically clamped in a vertical direction. The initial mechanical displacement corresponds to the stress free substrate in the whole model. The model is numerically solved by the finite element method with a time dependent solver in COMSOL 5.3. A triangular mesh with a maximal size 0.01 times the film thickness was used to obtain each result presented in the main text. The mesh element size outside the presented area decreased with a maximal rate of 1.1. Below are initial conditions and specific parameters for the numerical model settings used to obtain the presented results.

Figure 1: Film thickness 600 nm. Zero electric potential was set on the top and bottom surfaces of the film. A combination of regular and random oscillations was used as initial condition for polarization in the form:

$P_{x}(0)=0.3Rn(x)Rn(y)+0.7589\cdot{10}^{6}x(\cos\left( 2\pi\cdot{10}^{7}\left( x-y \right) \right))-{10}^{6}x(cos\left( 2\pi\cdot{10}^{7}\left( x+y \right) \right),$

$P_{y} \left( 0 \right)=0.3Rn(x+0.2)Rn(y+0.1)$,

where Rn(x) is a random seed function uniform from -1 to +1; hereafter y is measured from the film/substrate interface, x=0 corresponded to a position between the 4th and 5th CB from the left-hand side.

Figure 2: Film thickness 200 nm. Zero electric potential was set on the top and bottom surfaces of the film.

In Fig. 2a the following initial polarization values were set.

$P_{x}\left( 0 \right)= 0.7589\cdot\cos\left( 2\pi\cdot{10}^{7}\left( x-y \right) \right)$,

$\begin{matrix} P_{y}\left( 0 \right)= 0.739\cdot\cos\left( 3\pi\cdot{10}^{6}x \right) & P_{y}\left( 0 \right)= -0.739\cdot\cos\left( 7\pi\cdot{10}^{6}x \right) \\ x<250 nm & x\geq250 nm \end{matrix}$,

In Fig. 2b the polarization was set to the spontaneous value, pointing down everywhere except for a triangular region where it was set to the spontaneous value pointing right. The coordinates (x;y) for the triangle vertices are $(\times{10}^{-7}$): (3.3;1.5), (3.75;2) and (4.2;2). The four CBs has coordinates $x={n\cdot10}^{-7}$, where n=1,2,3,4.

Figure 3: Film thickness 20 nm. Zero electric potential was set on the bottom surfaces of the film. For Fig. 3a,b the top surface had potential $\varphi=2t\cdot{10}^{9}$, where $t$ is time. For Fig.3c,d the top surface potential was set constant according to indicated values. Initial conditions for polarization were set simply to induce the observed DWs. The four CBs has coordinates $x={n\cdot10}^{-8}$, where n=1,2,3,4.

# B: Energy for the crossing of ferroelastic a-domain with CB

Elastic energy ∆E of an a-domain passing through a CB is calculated using the method of fictitious dislocations. The CB is simply described as an effective dead layer (as shown in Fig. S1). Briefly, the fictitious dislocation method treats the system as if it were consisting of pieces with nominal lattice constants that are different in different domains but the same within each domain. The stresses and deformations are described in the frame of a linear theory as a sum of their fields coming from incompatibility defects, which are only present on domain boundaries. More details on the method in Refs. [32-35]. The schematic figure Fig. S1 illustrates the domains of three types: c-domain (unit cell nominally elongated vertically), a-domain (unit cell nominally elongated horyzontally), and the columnar boundary (unit cell nominally cubic). We are aiming to obtain expression for energy in terms of the following parameters: the width of the CB $d_{x}$ and the vertical shift of the a-domain $d_{y}$. It was shown that the part of the energy containing these parameters will be the same if the CB is ascribed nominal lattice constants the same as for c-domain. Hereafter, we consider only the incompatibilities corresponding to this simplified version. Their fictitious defects are edge dislocations, shown by symbols ˧ in Fig. S1. They give rise to the following elastic fields:

$\sigma_{xx}(b, x-x_{0}, y-y_{0})\equiv\sigma_{xx}(b, x^{'}, y^{'})=\frac{Eb}{4\pi(1-\nu^{2})}\frac{x^{'3}-3x^{'2}y^{'}-x^{'}y^{'2}-y^{'3}}{(x^{2}+y^{2})^{2}}$, (S7)

$\sigma_{xy}(b, x-x_{0}, y-y_{0})\equiv\sigma_{xy}(b, x^{'}, y^{'})=\frac{Eb}{4\pi(1-\nu^{2})}\frac{x^{'3}+x^{'2}y^{'}-x^{'}y^{'2}-y^{'3}}{(x^{2}+y^{2})^{2}}$. (S8)

In the context of our problem, $b\to\pm\varepsilon_{t}dy_{0}-$ different signs show on either side of the dead layer.

Following the theory of fictitious dislocations, the elastic energy $\triangle W(d_{y})$ is equivalent to the energy of interaction between dislocations. Thus, the effective force $f_{y}$ exerted by the set of dislocations at $x_{0}=0$ on the dislocations at $(d_{x}, y)$ in $y$-direction is given by

$$f_{y}\left( d_{x}, y \right)=\int_{-\frac{w}{2}}^{\frac{w}{2}} dy_{0}\left[ \sigma_{xx}\left( \varepsilon_{T}, d_{x}, y-y_{0} \right)+\sigma_{xy}\left( \varepsilon_{T}, d_{x}, y-y_{0} \right) \right]=$$

$\frac{E\varepsilon_{T}}{2\pi\left( 1-\nu^{2} \right)}\left( \frac{wd_{x}((w/2)^{2}+d_{x}^{2}-y^{2})}{(w/2)^{4}+2(w/2)^{2}(d_{x}^{2}-y^{2})+(d_{x}^{2}+y^{2})^{2}}-\mathrm{arctanh}\left( \frac{wy}{(w/2)^{2}+(d_{x}^{2}+y^{2})} \right) \right)$. (S9)

Then, the energy of displacing the row of dislocations at $x=d_{x}$ by $d_{y}$ in $y$ -direction can be found as

$\triangle W(d_{x}, d_{y})=-\int_{-w/2}^{w/2} \varepsilon_{T}dY\int_{Y}^{Y+d_{y}} dyf_{y}(d_{x}, y)$ (S10)

**Figure S1:** Crossing of the a-domain with CB. The treatment of the elastic subproblem in terms of fictitious dislocations. Only the relevant part of the defects is shown.

c

a

x

y

w

c

a

d

y

d

x

**Figure S3:** Crossing of the a-domain with CB. The treatment for electrostatic subproblem.

c

a

x

y

w

c

a

d

y

d

x

**Figure S2:** Numerically calculated elastic energy ∆*W*(*d_y_*) for various values of *d_x_/w* ratio: 0, 0.05, 0.1, 0.15, 0.2. As one can see, the minimum of elastic energy corresponds to *d_y_* ≈ *d_x_*.

0.1

0.2

0.3

0.4

0.5

-

0.2

-

0.1

0.0

0.1

0.2

Analytical expression for Eq. (S10) was found for the case $d_{x}=0$:

$\triangle W(0, d_{y})=\frac{E\varepsilon_{T}^{2}}{4\pi(1-\nu^{2})}(w^{2} ln |\frac{w^{2}-d_{y}^{2}}{w^{2}}|+2wd ln |\frac{w+d_{y}}{w-d_{y}}|+d^{2} ln |\frac{w^{2}-d_{y}^{2}}{d_{y}^{2}}|)$ (S11)

At $d_{y}\ll w$, this expression changes as $\propto d_{y}^{2}\ln d_{y}$. It has also been shown that the minimum of elastic energy is at $d_{y}\approx d_{x}$ at $d_{x}\ll w$ (see Fig. S2).

Further we calculated the electrostatic energy. To provide analytical treatment we used simplifications in analogy with the elastic theory above: homogeneous polarization inside each domain. As a result the mathematical treatment is similar, the role of fictitious dislocations is played by the density of bound charges, shown in Fig. S3. It is assumed that the bound charges are not compensated. For the calculation of the electrostatic energy we first derive the $y$-component of the electric field generated by bound charges at $x=0$ , which acts on the bound charges at $x=d_{x}$:

**Figure S4:** Numerically calculated electrostatic energy ∆*W*(*d_y_*) for different values of *d_x_/w* ratio: 0, 0.05, 0.1, 0.15, 0.2.

0.1

0.2

0.3

0.4

0.5

-

0.2

-

0.1

0.0

0.1

0.2

$E_{y}(d_{x}, y)=-\int_{-w/2}^{w/2} dy_{0}\frac{P_{S}}{\varepsilon_{b}}\frac{y-y_{0}}{d_{x}^{2}+(y-y_{0})^{2}}=\frac{P_{S}}{2\varepsilon_{b}}\ln\frac{d_{x}^{2}+(y-w/2)^{2}}{d_{x}^{2}+(y+w/2)^{2}}$ (S12)

Then, the energy of displacing the row of charges at *x* = *d_x_* by *d_y_* in *y*-direction can be found as

$\triangle W(d_{x}, d_{y})=-\int_{-w/2}^{w/2} P_{S}dY\int_{Y}^{Y+d_{y}} dyE_{y}(d_{x}, y)$*.* (S13)

The corresponding electrostatic energy is shown in Fig. S4. As one can see, the minimum of the electrostatic energy is always at $d_{y}=0.$ The electrostatic energy in case bound charges are partially compensated may be obtained by multiplication by a numerical factor. In Fig. 2 of the main text we use factor 1/300. The results presented in the main text correspond to $d_{x}=0$, $d_{y}=d$.

Note also that for the case $d_{x}=0$ used in the main text Eqs. (S9,S12) give essentially the same analytical expression up to a proportionality constant because $\mathrm{arctanh} \left( \frac{wy}{(w/2)^{2}+y^{2}} \right)=-\frac{1}{2}\ln\frac{(y-w/2)^{2}}{(y+w/2)^{2}}$. As a consequence, integrals in Eqs. (S10,S13) also produce the same expression, allowing us to write Eq. (2) of the main text in a form where it is very easy to separate electrostatic from elastic contribution to the energy penalty.

**C: Columnar structure in the thin films**


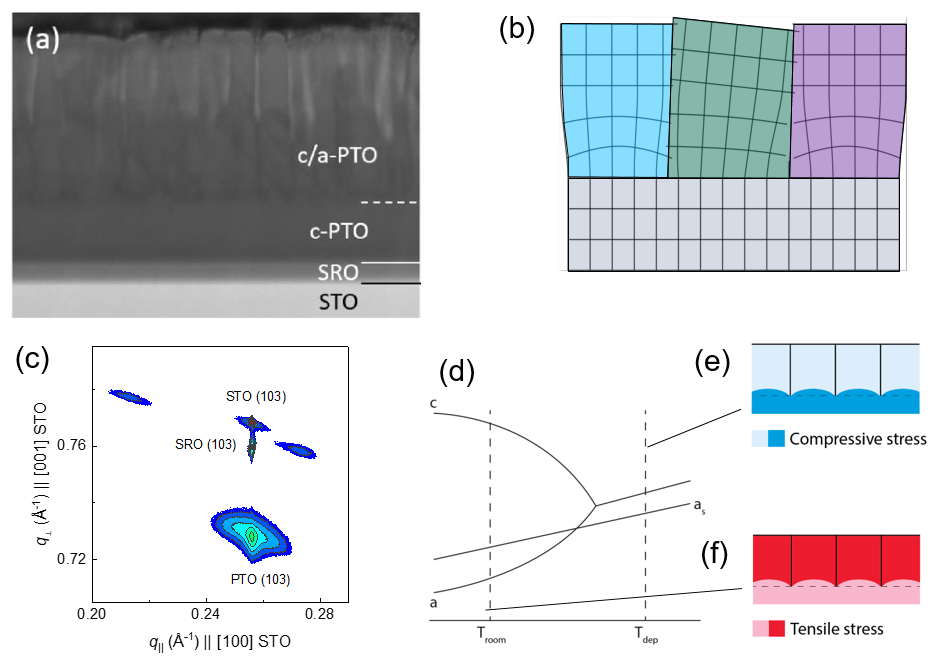
The supporting experiments on columnar films are carried out for the best studied perovskite oxide FE PbTiO_3_ (PTO), using the pulsed laser deposition (PLD) growth technique with a deposition temperature range 900 - 1000 K on (001) SrTiO_3_ substrate (STO) using bottom SrRuO_3_ (SRO) electrode layer and Pt top electrode pads. The columnar growth of such PTO film is ensured due to the large PTO-STO lattice misfit at the deposition temperature. The growth details can be found in Ref. [47]. The columnar structure is evidenced by a combination of X-ray diffraction analysis (XRD), and transmission electron microscopy (TEM). Fig. S5a shows the columnar structure in a 650-nm-thick film, grown at 973 K on (001) STO using a 60-nm-thick bottom SRO electrode. The TEM analysis reveals vertical columns in the PTO film, Fig. S5a. The CBs are well resolved at a distance larger than approximately 170 nm from the bottom PTO-SRO interface. This observation implies that the PTO film initially undergoes layer-by-layer growth. With increasing PTO thickness, 3D island growth gradually establishes and, at the thickness of 170 nm, CBs start appearing between islands.

**Figure S5.** Columnar structure in the thin film. (a) Cross-sectional TEM image. (b) – Schematic of the columnar structure and lattice defects at CBs. (c) XRD reciprocal space map around the (103) reciprocal lattice point (r.l.u. = 2π/*a_STO_* ≈ 16,09 nm^-1^). Maximum at h ∼ 0.96, l ∼ 2.96 corresponds to the c-domains, maxima at h ∼ 0.89, l ∼ 3.04 and h ∼ 1.12 and l ∼ 2.97 correspond to the tilted a-domains. (d)-schematics for the temperature dependence of the lattice constants in the thin film (a,c) and in the substrate a_s_. (e),(f) – schematics for the stress fields in the film at the deposition temperature (e) and after cooling prior to domain formation (f).

The driving force for the formation of the columns is the release of the compressive strain. Lattice orientations in the columns are schematically shown in Fig S5b, where in addition to deformations related to strain release, we assume spontaneous inclinations in each column due to the stochastic effects during growth. This way, column boundaries combine properties of stacking faults and low-angle twin boundaries. Fig. S5c evidence epitaxial cube-on-cube-type growth of tetragonal PTO containing the *c*- and *a*-domains, but not other crystal orientations of phases.

During post-deposition cooling, lattice constants in the substrate and in the film evolve according to the law schematically drawn in Fig. S5d. The associated evolution of the in-plane stress is schematically shown in Fig. S5e,f. Immediately after the ferroelectric transition, both *c* - and *a* - lattice constants of the film are larger than that of the substrate a_s_. This implies dominance of the c- ferroelastic domain with polarisation up or down at the high temperatures. On further cooling compressive strain for the c-domain changes to tensile, leading to inclusions of a-domains with polarization oriented in-plane of the film, which releases the tensile strain. During this process, the domain structure changes from one containing only 180-degree non-ferroelastic DWs, to another with both 180-degree DWs and ferroelastic 90-degree DWs.

Microscopic images in Fig. S6 provide further information on the columnar and domain structures in the PTO films. The CBs have distribution in the lateral size with a maximum between 50 and 100 nm. The cross-sectional images evidence that the ferroelastic a-domain inclusions are mainly present in the “columnar” part of the film. Only the largest a-domain penetrate into the lower “layer by layer” part of the film, and becomes narrower in this part. This observation is in agreement with the conceptual stress map shown in Fig. S5f. Indeed, the a-domains form to release the tensile stress. The higher stress in the “columnar” part gives rise to a higher a-domain fraction.

The fraction of a-domains can be estimated using Eq. (1) of the main text, which we repeat here

$\alpha=\left( 1+\nu\right)\frac{a_{s}-a}{c-a}$, (S14)

Using in Eq. (14) parameters for epitaxially grown PTO on STO ($a_{s}=0.3905$nm [29], $a=0.39045$, $c=0.41524$ [30]) give a fraction of the a-domains 0.2%, which is in agreement with our observation in the lower “layer by layer” part of the film (Fig. S6a). For the upper “columnar” part of the film the stress release due to CB formation at high temperatures must be taken into account. This part of the film has a mechanical state as if it were grown on a substrate with larger lattice constant. The experimentally observed domain fraction in the columnar part $\alpha$~ 15% may be readily interpreted using a larger effective substrate lattice constant $a_{s}=0.3933$ nm.

For the sake of simplicity in our phase field model we do not address the thickness-dependence of the stress relaxation, associated with the growth of CBs. We use the same effective substrate lattice constant $a_{s}=0.3934$ nm throughout the film (recalculated using the parameters from table S1 to obtain $\alpha$=0.15 in Eq. (S14)). This simplification extends the elastic properties of the main “columnar” part of the film to the smaller “layer by layer” part of the film. This explains why the simulated ferroelastic a-domains, Fig. 1b of the main text, occupy similar fraction of space in both “layer by layer” and “columnar” parts.


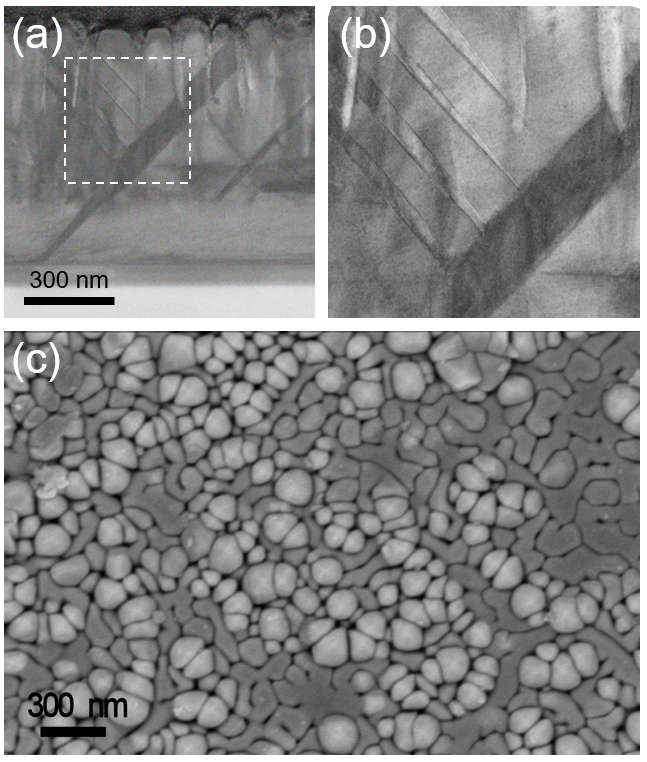


**Figure S6.** Columnar and domain structures in the thin film. (a) Cross-sectional TEM image. (b) – magnified part. (c)-SEM image showing the surface topography and the columnar structure.
